# Separation and Quantification of Eight Antidiabetic Drugs on A High-Performance Liquid Chromatography: Its Application to Human Plasma Assay

**DOI:** 10.5402/2011/521353

**Published:** 2011-07-12

**Authors:** Karunanidhi S. Lakshmi, Tirumala Rajesh

**Affiliations:** Department of Pharmaceutical Analysis, SRM College of Pharmacy, SRM University, Kattankulathur, Tamil Nadu 603203, India

## Abstract

An analytical method based on isocratic reverse phase high-performance liquid chromatography was developed and validated for the separation and quantification of eight antidiabetic drugs: rosiglitazone, pioglitazone, glipizide, gliclazide, repaglinide, nateglinide, glibenclamide, and glimepiride for their application in human plasma assay. Metformin is used as internal standard. Analysis was done on Onyx monolithic C_18_ column (100 × 4.6 mm, i.d., 5 **μ**m) using a mixture of 0.05% formic acid in water and methanol in the ratio of 42 : 58 (v/v) fixed at a flow rate of 0.5 mL/min, and they were monitored at 234 nm. Separation was achieved in less than 20 min. The calibration curves were linear in the range of 50–2000 ng/mL. The method was validated for its recovery, intra- and interday precision, stability, specificity, and selectivity. Plasma samples were prepared using solid-phase extraction of analytes. Hence, the developed method was found to be suitable for the routine analysis of selected antidiabetic drugs in biological matrices.

## 1. Introduction

**Figure 1:**
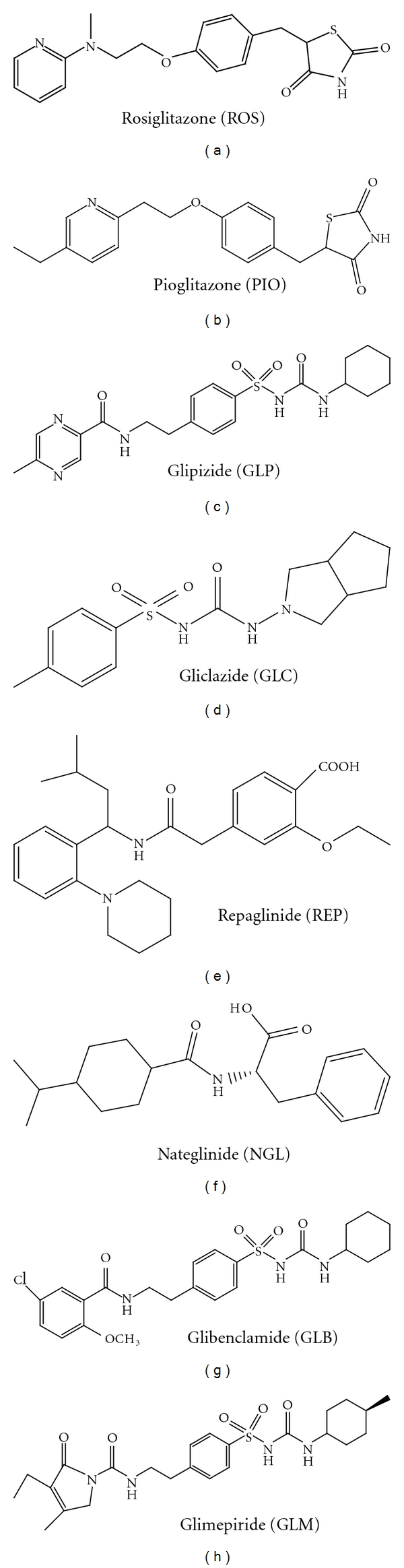
Structural representation of Rosiglitazone (ROS), Pioglitazone (PIO), Glipizide (GLP), Gliclazide (GLC), Repaglinide (REP), Nateglinide (NGL), Glibenclamide (GLB), and Glimepiride (GLM).

Diabetes mellitus is a heterogeneous group of disorders characterized by abnormalities in carbohydrate, protein, and lipid metabolism [1]. The central disturbance in diabetes mellitus is an abnormality in insulin production or action or both, although other factors can be involved. This results primarily in elevated fasting and postprandial blood glucose levels. In recent years, diabetes mellitus has become a common disease affecting human health seriously. There are estimated 150 million people worldwide sufferings from diabetes, this number may probably double by the year 2030. Reports from the World Health Organization (WHO) indicate that diabetes mellitus is one of the major killers of our time, with people in South East Asia and Western Pacific being most at risk. Therefore, the human population worldwide appears to be in the midst of an epidemic of diabetes. About 90% of diabetes patients are found to be affected with type II (non-insulin-dependent) diabetes mellitus. It is especially important to ensure the quality of antidiabetic drugs for type II in which insulin deficiency is less severe. All anti-diabetic drugs (structures shown Figures 1(a), 1(b), 1(c), 1(d), 1(e), 1(f), 1(g), and 1(h)) chosen in this study are commonly used in clinic for type II diabetes mellitus patients. Thiazolidinedione class of drugs (Rosiglitazone (ROS) and Pioglitazone (PIO)) exert their glucose-lowering effect by binding to peroxisome proliferator-activated receptors gamma (PPAR*γ*), thus increasing the receptor sensitivity to insulin [2–4]. Sulfonylurea drugs (Glipizide (GLP), Gliclazide (GLC), Glibenclamide (GLB), and Glimepiride (GLM)) act by increasing the secretion of insulin by the functioning *β* cells of the pancreas. This generation of hypoglycemic drugs are much more potent and are therefore effective at much lower dosages [5]. Repaglinide (REP) also acts by stimulating insulin secretion of *β* cells, but it binds to sites distinct from the sulfonylurea binding sites [6] and Nateglinide (NGL), functions by increasing pancreatic *β*-cell sensitivity to ambient glucose without increasing basal insulin secretion [7, 8]. 

In the literature survey, HPLC gradient method for six anti-diabetic drugs in human plasma [9], an isocratic method for counterfeit drugs in pharmaceutical formulations [10], and an HPLC method in human plasma for metformin with three sulfonylurea drugs glibenclamide, glipizide, and gliclazide [11] were reported along with few other LC-MS, HPLC, and HPTLC methods confined to single or two drugs in combination [12–27]. 

The present paper describes an isocratic method for the separation and quantification of eight anti-diabetic drugs using reverse phase HPLC-UV, as the usage of combination of thiazolidinediones and sulfonyl ureas was found to be successful in the treatment of type II diabetes, the method would help in assay of drugs in a single run which reduces the time of analysis and does not require separate methods for each drug. The developed method was also validated successfully applied for human plasma assay.

## 2. Materials and Methods

### 2.1. Chemicals and Reagents

PIO, GLP, REP, and Metformin (IS) were obtained from Macleoids Pharmaceuticals Ltd., Mumbai, India. GLC, GLB were obtained from Medley Pharmaceuticals, Diu and Daman, India. ROS and GLM were obtained from Orchid Chemicals and Pharmaceuticals, Chennai, India. NGL was obtained from Divis Pharmaceuticals, Hyderabad, India with more than 99% purity. Methanol (Qualigens, Mumbai, India) HPLC grade was used. SPE cartridges (Oasis HLB 3 mL, 60 mg, Waters, Milford, MA, USA) were used. All the reagents used were of analytical reagent grade. Milli-Q water (Millipore Q-Gard) was used throughout the analysis.

### 2.2. Instrumentation

The HPLC system consisted of Shimadzu (Kyoto, Japan) Class LC-10AT vp and LC-20AD pumps connected with SPD-10A vp UV-Visible detector. The data acquisition was performed by Spincotech 1.7 version software (Spinco Biotech Ltd., Chennai, India) and monitored at a wavelength of 234 nm. Samples were injected using 25 *μ*L Hamilton syringe.

### 2.3. Chromatographic Conditions

A reverse phase Onyx monolithic C_18_ column (100 × 4.6 mm i.d., 5 *μ*m, Phenomenex, Torrance, USA) was used for separation. The mobile phase consisted of 0.05% formic acid in water as solvent A and methanol as solvent B. A linear isocratic was run at 0.5 mL/min consisting of solvent A and B in the ratio of 42 : 58 v/v. The injection volume was 20 *μ*L.

### 2.4. Preparation of Standard and Sample Solutions

Stock solution of ROS, PIO, GLP GLC, REP, NGL, GLB, and GLM was prepared individually by dissolving 25 mg of each drug in methanol and diluted to 25 mL to get a final concentration of 1000 *μ*g/mL. A series of working standard solutions were prepared by transferring appropriate quantity in order to get a calibration range of 50, 100, 500, 1000, 1500, and 2000 ng/mL by using methanol. IS was prepared at a concentration of 1000 ng/mL in methanol.

### 2.5. Extraction of Drugs from Plasma

Solid-phase extraction was used to preconcentrate the analytes from the human plasma. All the samples were extracted from human plasma using Oasis HLB 3 mL, 60 mg (Waters, Milford, MA, USA), solid-phase extraction cartridges. Each cartridge was equilibrated and conditioned by acetonitrile, ultra pure water of 1 mL each. Samples were added to plasma and vortex-mixed for 3 min. Thereafter, the sample was loaded onto and passed through the cartridge without lab vacuum and the analytes were then eluted with 1 mL of acetonitrile. The eluent was evaporated to dryness at 40°C under a stream of nitrogen. The dried extract was then reconstituted with 100 *μ*L of mobile phase, and a 20 *μ*L was injected into the chromatographic system.

### 2.6. Method Validation

The proposed method was validated in the light of FDA Guidelines for linearity, precision, sensitivity and selectivity, stability and recovery [28, 29].

## 3. Results and Discussion

### 3.1. Development and Optimization of Chromatographic Conditions

Optimization of the chromatographic conditions are intended to take into account the various goals of method development and to weigh each goal (resolution, runtime, sensitivity, peak symmetry, etc.) accurately, according to the requirement of LC-MS and HPLC methods being used for the estimation of drugs in biological fluids.

**Figure 2:**
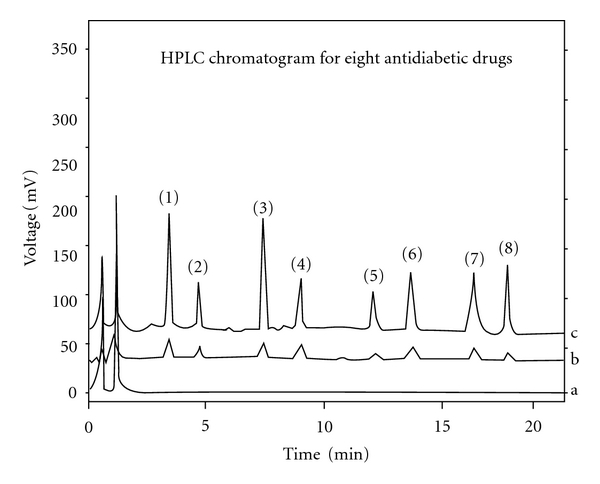
(a) Chromatogram showing blank, (b) chromatogram showing anti-diabetic drugs at LLOQ concentration levels, and (c) chromatogram showing drugs at 1000 ng/mL extracted from human plasma (1) ROS, (2) PIO, (3) GLP, (4) GLC, (5) REP, (6) NGL, (7) GLB, and (8) GLM.

The drugs are not totally soluble in water whereas soluble in organic solvents like methanol and acetonitrile. During the development phase, the mobile phase containing acetonitrile water resulted in asymmetric peaks with poor resolution and greater tailing factor (>2) and high run time. The successful use of mobile phase containing a mixture of 0.05% formic acid and methanol in the ratio of 42 : 58 v/v fixed at the flow rate 0.5 mL/min reduced tailing (<1.5) and resulted in good peak symmetry and resolution (>1.5). Methanol was selected because of its favorable UV transmittance, low viscosity, and better solubility. The analytes were monitored at 234 nm and the retention times were found to be 3.14, 4.58, 7.46, 8.95, 12.17, 14.39, 16.32 and 18.78 min for ROS, PIO, GLP, GLC, REP, NGL, GLB, and GLM, respectively, and IS was eluted at 2.36 min (Figure 2).

### 3.2. Validation of the Developed Method

#### 3.2.1. Linearity and Calibration Curve

The linearity was tested at the concentration range of 50–2000 ng/mL and the calibration curve constructed was evaluated by its correlation coefficient. The correlation coefficient (*r* ^2^) for all the calibration curves was consistently greater than 0.9984 ± 0.0006 (Table 1).

#### 3.2.2. Method Sensitivity and Specificity

Within the same 30-min LC run, all the drugs at 50 ng/mL each under investigation could be easily detected from human plasma matrices (*n* = 10). Confirmation of these drugs could be readily achieved by comparing the retention times obtained from the sample with those of their corresponding drug standards. The lower limit of quantification (LLOQ) (Table 1) was defined as the lower concentration that could consistently produce accurate and precise chromatogram that could be quantified. Figure 2-b shows the chromatogram of the drugs obtained from plasma and samples spiked with the concentration at LLOQ. 

The method specificity was assessed with different plasma samples (*n* = 30 each, each from different plasma sources) analyzed with the described method. Interferences from the matrices at the targeted retention times were not observed.

#### 3.2.3. Precision and Accuracy

The intraday precision (expressed by coefficient of variation of replicate analyses) was estimated on the three quality control levels and the interday precision on the nine calibration standard levels.  Table 2 shows the results obtained for the intraassay (variation intraday) and inter-assay (variation inter-day) precision for drugs. The precision for all these analytes under investigation did not exceed 15% at any of the concentrations studied and well met the requirements of validation.

#### 3.2.4. Stability

The stability of samples reconstituted after extraction from plasma (as in experimental section) was investigated under various storage conditions. Short-term stability was studied at room temperature for 8 h, long-term stability studies were done at −70°C for 30 d, and freeze-thaw stability was also evaluated by successive cycles of freezing and thawing the samples by storing at −70°C and room temperature, respectively. Three complete freeze-thaw cycles were performed. They were carried out at two concentration levels (low and high QC) at six replicates. The percentage stability was estimated by comparing the mean of back calculated concentrations of all analytes from the stored stability samples with that of freshly spiked QC samples. The results indicated that each analyte had an acceptable stability under those conditions, as shown in Table 3.

### 3.3. Recovery of Drugs from Human Plasma

Solid-phase extraction technique was found to be successful in extraction of eight anti-diabetic drugs from human plasma and the recovery was determined by comparing peak areas of spiked plasma extracts with those of unextracted neat standards freshly prepared in methanol. Plasma samples (*n* = 6) spiked with the analytes at their respective LLOQ, low, middle and high QC levels were analyzed. The area ratios of the targeted drugs were compared with those obtained from blank extracts spiked with the 8 target drugs after extraction (taken as 100% recovery of the drug from that particular matrix). Recoveries of the drugs are summarized in Table 4.

## 4. Conclusion

A simple, specific, selective, and precise method was developed for the determination of anti-diabetic drugs ROS, PIO, GLP, GLC, REP, NGL, GLB, and GLM. The mobile phase is economical and simple to prepare with little or no variations. The run time 30 min indicates short analysis time. The sample recoveries in human plasma were in good agreement and they suggested no interference in the estimation. Hence, this method can be easily and conveniently used for the routine analysis of the drug in plasma samples for pharmacokinetic studies.

## Figures and Tables

**Table 1:** Results of the linearity, LLOQ, and system suitability parameters (*n* = 3).

Compound	*λ*_max⁡_	LLOQ (ng/mL)	*r* ^2^	*t*_*r*_	*k*′	*R*_*s*_	*T*_*f*_	*W*_(5%)_	*N*	HETP (mm)
ROS	245	24	0.9983	3.14	2.31	—	1.17	0.2876	9945	0.0101
PIO	265	36	0.9981	4.58	4.59	2.42	1.09	0.2912	9233	0.0108
GLP	270	35	0.9989	7.46	2.17	5.52	1.34	0.2764	10637	0.0094
GLC	225	30	0.9990	8.95	2.72	3.14	1.27	0.2819	7425	0.0135
REP	245	20	0.9987	12.17	3.11	7.01	1.25	0.3011	9910	0.0101
NGL	231	20	0.9989	14.39	2.79	3.47	1.42	0.3082	8941	0.0112
GLB	228	40	0.9978	16.32	2.09	7.46	1.37	0.2941	10003	0.0099
GLM	246	38	0.9987	18.78	2.57	2.89	1.28	0.2659	9081	0.0110

*t*_*r*_: retention time; *k*′: capacity factor; *R*_*s*_: resolution; *T*_*f*_: tailing factor; *W*_5%_: width of peak at 5%; *N*: theoretical plates (USP); HETP: height equivalent to theoretical plate Length of the column (mm)/theoretical plates.

**Table 2:** Summary of precision and accuracy from QC samples in plasma (*n* = 6).

Compound	spiked concen.	Intraday			Interday		
	(ng/mL)	Mean ± SD	%RSD	Nominal (%)	Mean ± SD	% RSD	Nominal (%)
	53.7	53.4 ± 3.7	6.93	99.4	53.0 ± 3.6	6.79	98.6
ROS	489.2	481.6 ± 19.9	4.13	98.4	481.9 ± 20.7	4.29	98.5
	1531.0	1520.5 ± 129.5	8.51	99.3	1521.7 ± 117.8	7.74	99.3

	48.9	48.6 ± 3.5	7.20	99.4	48.1 ± 2.9	6.02	98.3
PIO	491.2	490.5 ± 16.2	3.30	99.8	492.0 ± 15.9	3.23	100.1
	1513.8	1492.3 ± 132.9	8.90	98.5	1497.0 ± 125.6	8.39	98.8

	50.6	52.1 ± 3.3	6.33	102.9	50.9 ± 4.0	7.85	100.6
GLP	492.3	504.7 ± 18.9	3.74	102.5	499.3 ± 19.9	3.98	101.4
	1498.5	1506.1 ± 109.2	7.25	100.5	1500.1 ± 130.2	8.67	100.1

	46.8	48.2 ± 3.9	8.09	102.9	48.2 ± 4.1	8.50	102.9
GLC	486.7	492.9 ± 15.8	3.20	101.2	490.8 ± 18.5	3.76	100.8
	1467.0	1479.0 ± 119.2	8.05	100.8	1475.6 ± 125.6	8.51	100.5

	49.2	51.1 ± 2.9	5.67	103.8	48.9 ± 3.8	7.77	99.3
REP	493.4	486.8 ± 15.9	3.26	98.6	487.1 ± 17.3	3.55	98.7
	1502.7	1489.4 ± 123.7	8.30	99.1	1486.9 ± 91.7	6.16	98.9

	52.9	51.7 ± 3.1	5.99	97.7	51.5 ± 3.0	5.82	97.4
NGL	478.6	492.5 ± 17.2	3.49	102.9	490.2 ± 16.9	3.44	102.4
	1439.6	1452.4 ± 134.1	9.23	100.9	1442.3 ± 121.3	8.41	100.1

	45.7	45.4 ± 3.2	7.04	99.3	44.9 ± 4.1	9.13	98.2
GLB	478.5	481.3 ± 28.8	5.98	100.6	479.6 ± 20.9	4.35	100.2
	1452.0	1450.3 ± 110.1	7.58	99.8	1451.9 ± 97.9	6.74	99.9

	48.6	49.1 ± 2.9	5.90	101.0	48.5 ± 3.5	7.21	99.7
GLM	490.5	499.6 ± 25.6	5.12	101.8	492.8 ± 24.3	4.93	100.4
	1477.5	1479.0 ± 121.1	8.18	100.1	1470.8 ± 115.9	7.88	99.5

**Table 3:** Stability data for anti-diabetic drugs in plasma.

Drug	Nominal concentration	Sample conditions (mean ± S.D)					
	ng/mL	Short-term^a^		Long-term^b^		Freeze-thaw^c^	
		Nominal (%)	RSD	Nominal (%)	RSD	Nominal (%)	RSD
ROS	53.7	103.1	6.2	97.5	8.6	102.4	7.1
	1531.0	99.3	2.0	98.0	2.2	98.7	1.7

PIO	48.9	102.0	5.9	97.1	8.8	100.6	5.9
	1513.8	99.0	2.0	97.2	1.9	99.1	1.8

GLP	50.6	98.9	7.6	95.6	8.0	97.1	6.6
	1498.5	99.3	1.5	98.7	1.4	99.8	0.9

GLC	46.8	105.7	8.7	99.6	3.4	100.1	2.3
	1467.0	97.8	2.0	97.3	4.1	98.1	4.5

REP	49.2	107.8	8.9	99.2	3.8	99.2	3.0
	1502.7	103.5	6.1	98.1	0.8	98.6	1.4

NGL	52.9	98.9	11.1	96.7	7.2	96.9	5.3
	1439.6	108.0	6.4	99.0	4.8	98.5	2.8

GLB	45.7	95.4	13.2	95.1	10.6	95.5	7.8
	1452.0	97.8	10.1	96.8	6.8	96.2	5.9

GLM	48.6	103.7	11.6	99.1	1.9	98.7	2.2
	1477.5	106.3	6.0	98.7	2.3	98.6	1.0

^a^Exposed at room temperature for 24 h.

^b^Stored at −70°C.

^c^After three freeze-thaw cycles.

**Table 4:** Recovery of eight anti-diabetic drugs from plasma samples spiked with each drug spiked.

Compound	Concentrations (ng/mL)				% recovery and % RSD			
	LLOQ	LQC	MQC	HQC	LLOQ	LQC	MQC	HQC
ROS	24.3	53.7	489.2	1531.0	80.1, 9.1	77.2, 11.3	81.5, 7.2	83.4, 6.9
PIO	36.1	48.9	491.2	1513.8	82.4, 7.3	80.6, 10.1	81.9, 8.1	82.3, 7.2
GLP	35.6	50.6	492.3	1498.5	80.9, 6.7	81.9, 14.7	82.3, 7.7	84.0, 9.8
GLC	30.5	46.8	486.7	1467.0	79.9, 5.9	84.5, 12.6	83.2, 11.1	79.7, 13.9
REP	20.2	49.2	493.4	1502.7	84.5, 4.9	80.8, 14.2	80.9, 8.7	82.4, 9.7
NGL	20.6	52.9	478.6	1439.6	80.8, 8.8	83.4, 13.7	82.1, 12.4	80.9, 10.6
GLB	40.1	45.7	478.5	1452.0	79.1, 10.3	75.5, 12.2	78.9, 12.7	76.8, 12.3
GLM	38.4	48.6	490.5	1477.5	81.2, 10.7	78.2, 11.0	80.1, 4.7	80.1, 10.
